# Women’s perspectives of the fetal fibronectin testing process: a qualitative descriptive study

**DOI:** 10.1186/1471-2393-14-190

**Published:** 2014-06-04

**Authors:** Wendy E Peterson, Ann E Sprague, Jessica Reszel, Mark Walker, Deshayne B Fell, Sherry L Perkins, Sandra I Dunn, Moya Johnson

**Affiliations:** 1 School of Nursing, Faculty of Health Sciences, University of Ottawa, 451 Smyth Road, Room 1118 F, Ottawa, Ontario K1H 8M5, Canada; 2 BORN Ontario (Better Outcomes Registry & Network), Ottawa, Canada; 3 Children’s Hospital of Eastern Ontario Research Institute, Ottawa, Canada; 4 Department of Obstetrics and Gynecology, Faculty of Medicine, University of Ottawa, Ottawa, Canada; 5 Ottawa Hospital Research Institute, Ottawa, Canada; 6 Department of Pathology and Laboratory Medicine, University of Ottawa and The Ottawa Hospital, Ottawa, Canada

**Keywords:** Fetal fibronectin, fFN, Screening, Preterm birth, Preterm labour, Symptoms, Maternal experience

## Abstract

**Background:**

In 2009 the Ontario Ministry of Health and Long Term Care funded the implementation of province-wide fetal fibronectin testing in Ontario hospitals. This paper reports results from the provincial evaluation that sought to describe the experience of fetal fibronectin testing from the perspective of women with symptoms of preterm labour.

**Methods:**

A descriptive qualitative design was used, employing semi-structured telephone and face-to-face interviews with women who had fetal fibronectin testing.

**Results:**

Five hospitals participated in recruiting women for the study and 17 women were interviewed. Women described their experiences of fetal fibronectin testing as an emotional process that moves from expecting, to feeling, to hoping for reassurance; and then to re-defining what is required to feel reassured. Women described feeling anxious while waiting for fetal fibronectin results. When test results were negative, women described feeling a sense of relief that their symptoms would not likely lead to an imminent preterm birth. Women with positive results expressed feeling reassured by the care decisions and quick action taken by the health care team.

**Conclusion:**

Fetal fibronectin testing was acceptable and beneficial to these women with symptoms of preterm labour. Implications for practice and future research are suggested.

## Background

Preterm birth (PTB) (prior to 37 weeks gestation [1]) is a leading cause of neonatal morbidity and mortality and results in costly neonatal intensive care and long-term negative health outcomes. While some PTB is the result of induction of labour or caesarean section for maternal or fetal indications, spontaneous labour accounts for the majority of PTBs [1-3]. In Canada approximately 20,000 (5.5%) newborns per year are born prematurely as a result of spontaneous preterm labour (PTL) [2]. The rate is similar in Europe and slightly higher in the United States (6%) [4].

The medical and nursing care required for preterm infants is specialized and costly. Neonatal outcomes are improved when women in PTL are transferred from community hospitals to those with neonatal intensive care units (NICUs) prior to birth [5]. In 2006, two health system problems in Ontario, Canada were identified. First, there was a concerning increase in the number of women giving birth to preterm infants in facilities without the appropriate level of neonatal care. And secondly, more women with symptoms of PTL were being transferred to hospitals out of region, province and country (to the United States) and given the unpredictable nature of PTL, many of these women were transferred back home still pregnant [6].

Fetal fibronectin (fFN) is a glycoprotein that, when absent in cervicovaginal secretions between 24–34 weeks gestation, indicates that a woman is unlikely to give birth within the next 7–14 days [7]. The test, using a vaginal swab, is easy to perform and test results are rapidly available.

Given the excellent negative predictive value of the fFN screening test [8], and the anticipated cost savings in avoiding unnecessary transfers, the Provincial Council for Maternal and Child Health recommended provincial implementation of fFN testing for women presenting at 24–34 weeks gestation with PTL symptoms. In 2009 the Ministry of Health and Long Term Care funded the universal implementation and efficacy evaluation of fFN testing in all Ontario hospitals [9,10].

While the clinical value of fFN testing has been studied, it is also important to understand how women experience fFN testing. Exploring fFN testing from the women’s perspective is essential to our comprehensive understanding of the test’s potential benefits and harms [11]. To date, very few studies [12,13], and no qualitative studies, have examined the experience of fFN testing from the perspective of women with symptoms of PTL. The purpose of this study was to answer the research question: how do women with symptoms of PTL describe their experience of fFN testing?

## Methods

A qualitative descriptive method was employed. Qualitative description is appropriate when the researchers’ intention is to inductively derive a description of a particular phenomenon about which little is known. Data are commonly transcribed text from interviews with individuals who have experienced the phenomenon of interest. Although researchers using qualitative description produce an interpretation of the data, there is relatively less depth to the interpretation than studies employing a phenomenological or grounded theory approach [14,15]. Therefore the findings of qualitative descriptive studies are described as remaining “closer to the data” [16] and are appropriate for exploratory purposes. The study was approved by the Research Ethics Boards of The Ottawa Hospital and each of the hospitals where participants were recruited.

### Setting

Nine Ontario hospitals were invited to recruit participants for the study. These hospitals were purposefully selected to reflect a mix of different levels of maternal-newborn care and rural, remote and urban populations. Two hospitals declined to participate due to very low volumes of women presenting with symptoms of PTL. The research coordinator identified a contact person at each of the remaining seven hospitals who agreed to facilitate recruitment.

### Participants

Women were eligible to participate if they presented to one of the seven hospitals with symptoms of PTL, received the fFN test, and spoke English or French. Eligible women were introduced to the study by care providers involved in administering the fFN test. An information sheet about the study was wrapped around each fFN test kit to remind providers to introduce and determine interest in the study. The study coordinator contacted site coordinators weekly to obtain a list of interested participants. Women were contacted by telephone by one of three research assistants (two Anglophone, one Francophone) within one week of indicating an interest in the study. During this initial telephone conversation, the research assistant described the purpose and data collection methods of the study and invited the woman to participate. A consent form was mailed to interested participants for their signature. Once the signed consent form was returned to the research team by mail, an interview was scheduled at a mutually convenient time.

### Data collection

Between November 2010 and June 2011 the research assistants (RAs) conducted 1:1 digitally recorded interviews with participants. The RAs were all registered nurses studying at a graduate level with training and previous experience in conducting qualitative interviews with pregnant and parenting women. Women living in the same city as the RA were offered the choice of a face-to-face or telephone interview. Prior to the start of each interview the RA reviewed the study purpose and procedures, answered participants’ questions, and received verbal confirmation of participants’ informed consent. The semi-structured interview guide included eleven structured questions about the fFN test (e.g. results; subsequent admission or transfers) and seven open-ended questions inquiring about their experiences having the fFN test (Table 1). The use of semi-structured interviews facilitated discussion on predefined topics while allowing the flexibility for the women to speak freely on the subject [17].

**Table 1:** Interview guide

1	Please tell me about your pregnancy.
2	Tell me about the circumstances that led up to you having the fFn test?
3	How was the fFN test explained to you?
4	What did the fFN test results mean to you?
5	How confident were you about the results of this test in helping to determine if the baby would be born early or not?
6	Based on your experience, would you change anything about the care you received?
7	In reflecting on your experience, how did having this test make a difference in your life?

### Data analysis

Following each interview, the digital recordings were transcribed verbatim by a transcriptionist and identifying information removed. The transcripts were verified by the RAs to correct errors and entered into NVivo8 for data management. Data analysis was conducted by two researchers (WEP, JR) and followed the conventional content analysis approach described by Hseih and Shannon [14]. This inductive process involved (1) an initial independent reading of transcripts with notes made in margins by each of the two researchers, (2) re-reading transcripts and joint development of a preliminary coding scheme describing key thoughts (tables were constructed listing the codes, descriptions, and examples), and (3) continued re-reading and ongoing discussions to reach consensus about common patterns and groupings of codes into themes and sub-themes that describe the women’s experiences. Data saturation was assessed throughout sampling, data collection, and data analysis. No further participants were recruited once no new themes emerged from the data [18].

## Results

Five of the seven participating hospitals recruited women for the study. In total, thirty-seven women were eligible to participate. Of the 37 eligible women, 4 (11%) refused to have an RA phone them to tell them about the study, 9 (24%) women could not be contacted by the RA (did not return messages; wrong number), 2 (5%) women refused to participate after speaking with the RA, and 5 (14%) women did not attend their scheduled interviews. A total of 17 women participated in an interview between November 2010 and June 2011.The interviews were conducted within 9 days to 2 months after the women’s fFN test and were approximately 30 minutes long; two were conducted in person and 15 by telephone. Fifteen interviews were conducted in English and two in French. Three of the seventeen women had given birth by the time of their interview (two at < 32 weeks; one at term). Ten participants were having their first baby and seven women already had at least one child. Most participants lived in urban areas, had some college or university education, and were employed (Table 2). Eight participants were fFN positive, six were fFN negative, and two women did not know their results at the time of their interview. One woman had the fFN screening test three times throughout her pregnancy, screening fFN positive at 27 weeks and negative at 29 and 30 weeks gestation. None of the women had had a previous PTB.

**Table 2:** Characteristics of participants (N = 17)

**Characteristic**	**N (%)**
At time of interview	
Pregnant (range 26–36 weeks)	14 (82.4)
Postpartum (range 3–5 weeks)	3 (17.6)
Maternal age	
≤ 24	3 (17.6)
25-34	11 (64.7)
35-44	3 (17.6)
Number of children (range 0–2)	
0 (first pregnancy)	10 (58.8)
1	4 (23.5)
2	3 (17.6)
Marital status	
Single with partner support	4 (23.5)
Married	13 (76)
Residence	
Urban Ontario	15 (88.2)
Rural Ontario	2 (11.8)
Education	
Some high school	2 (11.8)
Completed high school	2 (11.8)
College/University	13 (76.5)
Employment	
Currently employed (including maternity leave)	15 (88.2)
Currently not employed	2 (11.8)
Hospital	
A	4 (23.5)
B	4 (23.5)
C	1 (5.9)
D	3 (17.6)
E	5 (29.4)

The most common reason women gave for presenting at a birthing unit was that they had been advised to do so during a telephone conversation or in-person visit with a health care professional (n = 9). More specifically, three women sought telephone advice from registered nurses via a provincial telephone health service (Telehealth), three spoke with or visited their primary provider, and another three telephoned their birthing unit directly. In all cases the women were advised to be seen in hospital immediately. Seven participants made their decision to seek care independently or with their partner, and one woman went to the birthing unit on advice of a work colleague who was also pregnant. Most women described arriving at the birthing unit feeling uncertain about the need to seek care urgently, expecting that their symptoms were not indicative of PTL and to be reassured that all was well.

Women’s experiences of fFN testing fit within the overarching theme of ‘seeking reassurance’. This overarching theme encompasses three other themes: (1) feeling reassured by being assessed in a birthing unit, (2) hoping for reassurance from the test, and (3) re-defining reassurance after learning the results. These three themes are further divided into sub-themes that support and explain this interpretation of the women’s experiences (Table 3). In the following paragraphs we present selected quotes to illustrate the themes and their respective sub-themes.

**Table 3:** Organization of themes

**Overarching theme**	**Themes**	**Sub-themes**
Seeking reassurance	1. Feeling reassured by being assessed in a birthing unit	1.1 Birthing unit environment
		1.2 Reassuring talk
	2. Hoping for reassurance from the test	2.1 Needing clear explanations
		2.2 Needing a support person while waiting for the results
	3. Re-defining reassurance after learning the results	3.1 Negative fFN testing results
		3.2 Positive fFN testing results

### Feeling reassured by being assessed in a birthing unit

In this first theme, women described how the process of being tested for fFN contributed to feeling reassured and the essential elements required for the experience to be reassuring: the birthing unit environment and reassuring talk.

#### ***The birthing unit environment was a reassuring place for participants***

Three characteristics of the birthing unit were described as contributing to the women’s sense of reassurance: multiple assessments and tests, the busy nature of the birthing unit, and confidence and trust in the birthing unit team.

As part of the assessment for PTL, women underwent ***multiple assessments and tests ***of which fFN testing was only one. Women recounted being placed on fetal monitors, having cervical exams, blood work, ultrasounds and other procedures as outlined by this participant:

*“They asked me… my past history with my health. They took my blood pressure…They kept monitoring my blood pressure and then they did a urine sample and a whole bunch of blood work. And then that’s when they asked if they could do the - that [fFN] test thing. And I said, “Yes, fine”. And they did that and then they did… They checked to see if I was dilated which I wasn’t….They were very happy with the fetal monitor…the heart beat and how active she was and everything so I got sent home after”.* (Participant 17)

The women’s experiences of having multiple tests and assessments in the birthing unit contributed to their sense of reassurance.

***The busy nature of the birthing unit ***was clearly noticed by participants. Interestingly, women interpreted their own experience within the busy units as reassuring whether or not they had been triaged as a priority. One woman remarked how she could overhear other women in labour and staff caring for them while she waited to be seen. She explains that overhearing the busy unit activity and other women in labour, was reassuring.

*“I could realize… there’s other women in more serious conditions than me, you know, women that are actually [in] full blown, going into labour…preterm… 30 weeks. I could hear what was going on around in the other rooms…It also made me feel better too that they didn’t think they needed to rush with me”.* (Participant 1)

Alternatively, women also felt reassured when they had been triaged as the priority case.

*“Once I got* [to the hospital] *I had a dedicated nurse right from the get go, so a nurse was always there. And then once they said that it was a positive result they just checked me more often but they were, I still had that dedicated nurse and that care”.* (Participant 14)

Most women expressed a sense of reassurance from the ***confidence and trust ***that they had in the birthing unit team.

*“And I think a big part of being a woman and…getting obstetrical care is a trust component … You know, being able to say, this person has more experience than me, this person has seen much more than me and I know that when push comes to shove, they will be… Have me and my health and the health of my child at their heart and at their best interest, and I know with complete certainty that I don’t have to…second guess what they’re saying, that I can just wholeheartedly agree with what they’re doing”.* (Participant 4)

Participants were clearly reassured by being assessed for PTL in a birth unit environment. Regardless of whether the women were triaged as a priority or not, the experience of being assessed in a busy unit by specialized practitioners was reassuring.

#### ***Reassuring talk***

The second sub-theme of women’s fFN testing process that contributed to their reassurance was the reassuring talk of the nurses and physicians.

*“Everyone who I talked to, including the neonatal doctor said that 32 weeks is a good age, you know, they most, 98% or whatever statistics they gave me is most babies are okay. But he explained that you know, he might have breathing difficulties and he explained what happens during the birth when they… So that was kind of reassuring, I wasn’t actually that worried … I mean I didn’t want to have the baby but I kind of felt comforted by the fact, okay these medical people know what they’re doing”.* (Participant 3)

This reassuring talk from the staff in the birthing unit throughout the women’s assessment contributed to their experience of reassurance.

### Hoping for reassurance from the test

As women talked about their experiences there was a common theme of moving from ‘feeling reassured’ by the birthing unit environment to ‘hoping for reassurance’ that they were not in PTL. In combination with the other assessments that women underwent, the availability of the fFN screening test contributed to their hope for reassurance. Two sub-themes emerged as important conditions for maintaining hope: clear explanations and the need for a support person while waiting for results.

#### ***Needing clear explanations***

Only two women had heard about fFN testing prior to their test. Women identified having the fFN screening test explained to them as an important condition to their experience of hoping for reassurance. One woman explains the importance of understanding the meaning of results in order for women to perceive value in fFN testing.

*“I think the value of the test kind of directly relates to probably how well it’s explained to people, you know, and if they’re not given a really good explanation of what the test is then it doesn’t have as much value for the patient…”* (Participant 6)

The following quote is illustrative of most of the women’s understanding of fFN testing.

The doctor *“… just explained that it was…a swab that they were doing of the cervical area and that it would provide us with information regarding whether labour* [and birth] *is imminent within the next two weeks”.* (Participant 9)

This participant continues with words that reflect her hope for reassurance from the fFN results.

*“And if …it came back negative,* [then] *that proved that there was no issue…regarding the labour, that way we didn’t have to worry. And if it came back positive then they would have to do further tests and monitor us further”.* (Participant 9)

Receiving a clear explanation of the fFN test and understanding the consequences of specific results facilitated the women’s experience of hoping for reassurance from the results of the test.

#### ***Needing a support person while waiting for the results***

Most of the participants were either thankful that they had a support person with them or expressed the wish that their support person had remained with them while they waited for test results. When women described their experience waiting they spoke of feeling increasingly anxious. For example, one participant explains that her husband *“…was holding my hand through the whole test, waiting for the test results just like I was, and just as anxious as I was”* (Participant 16). Another woman explains that *“…waiting for the test results was really, really anxious…”* (Participant 1). When this participant was asked what could be done differently she replied *“Probably make* [my husband] *stay, if it ever happened again make him stay with me through the whole thing”* (Participant 1).

Another participant agreed that having had her husband present when she received the fFN results would have helped:

*“Because I rely on him for all the medical terms kind of thing, because I find sometimes, you know, the doctors and nurses get so caught up with all the medical terms that they start speaking to you and it’s like, ‘uh hello? I didn’t understand a single word that’s coming out of your mouth’…all I heard was ‘you’re okay’ …So anything else other than that…I wasn’t really paying attention…I was on morphine too… I was really out of it”.* (Participant 2)

The presence of a support person during this period of hoping for reassurance from the test results and when women received their results was commonly described as essential.

### Re-defining reassurance after learning the results

Being informed of the fFN results marked the beginning of a period of time where women re-defined what they would consider to be reassuring. The following quotes are used to illustrate how women with negative and positive fFN testing results re-defined what would be required to feel reassured.

#### ***Negative fFN testing results***

Women with negative fFN results expressed feeling reassured by knowing that they are not likely to give birth within the next 7–14 days. For example:

*“…it was reassuring to know that we likely wouldn’t have our baby in the next two weeks, so that was sort of a positive in just sort of knowing that anything that might be happening to my body was maybe manageable and might still be a red flag but just to know that it probably wasn’t labour or preterm labour. So it sort of just eased my mind that way”.* (Participant 15)

The women’s feelings of reassurance were furthered by their sense of the health care providers’ confidence in the meaning of a negative result.

*“It was as soon as I got the results back that they* [the doctors and nurses] *were like, ‘okay, it’s okay to go home… you shouldn’t go into labour for seven days’ and it was their confidence [that] made me more confident in it as well”.* (Participant 7)

Importantly, although women were initially reassured that PTL was not imminent they ‘re-defined’ what was now required to reassure them by expressing concerns about the cause of their symptoms.

*“And I still didn’t have a kind of full diagnosis of what was going on with me. I did get treated for a bladder infection but she wasn’t entirely convinced that’s what it was but it was the only thing that they could really treat for at the time”.* (Participant 15)

*“But still at the same time, I wanted to know what caused the problem, what was behind it…They said they didn’t know what was wrong”.* (Participant 2)

While the women who received negative fFN results were reassured that they were not going to give birth in the next 7 to 14 days, their need for reassurance was commonly re-defined through the ongoing questions about the cause of their PTL symptoms.

#### ***Positive fFN testing results***

Interestingly, women with positive fFN results also re-interpreted what they found reassuring. After receiving their results, women described feeling reassured by the speed with which care decisions were made. Decisions included hospital admission or transfer, consultations and administration of steroids to enhance fetal lung maturity.

“*And so then it just kind of felt like, ‘okay this* [test result] *means something’ because as soon as they said the results are positive, I heard them on the phone with my family physician and they said they were going to admit me*”. (Participant 3)

Once their symptoms stabilized, some women with positive screening results re-defined what would provide them with reassurance yet again. In the following two examples women have moved away from being reassured by the health care team’s attention and care to needing an understanding about the cause of their PTL.

*“I’m happy but I’m a little bit nervous to be home and not be watched. I’m still having contractions and they’re not really telling me why*”. (Participant 10)

*“I just keep being told that they don’t really know why because they keep ruling out the very few reasons that women do go into preterm labour”.* (Participant 16)

Upon learning their positive fFN test results, the women described feeling reassured by the speed of the care decisions made by the health care team. Like women with negative fFN results, once women with positive fFN results were stabilized, they identified that they would feel reassured by knowing the cause of their PTL.

## Discussion

This paper reports the findings from a study of women who presented to birthing units for assessment of symptoms of PTL. Unlike other studies of women’s experiences with fFN testing, the women in this study had not been previously identified as high risk or diagnosed with PTL. Therefore, this study contributes novel in-depth understanding of women’s experiences with fFN testing from the onset of PTL symptoms.

Study participants experienced a variety of symptoms of PTL and expressed feeling uncertain about the need to seek health care. Similarly, other studies have concluded that PTL is often not within expectant women’s consciousness, and may result in delays seeking care [19,20]. After some delay in seeking care, nine of the women in this study sought the advice of a health care professional, which is consistent with findings from Kingston and Chalmers [21] who found that health care providers were considered to be the most important source of information in pregnancy by Canadian women. Consistent with current practice recommendations, the registered nurses, physicians and midwives consulted by the women in this study all advised the women to go to the birthing unit for an immediate assessment [22,23]. Since vaginal swabs for fFN screening are processed in hospital and must precede a digital exam for dilatation, providers may be more likely to advise women to proceed directly to hospital for an assessment than previously when initial assessments could be conducted in physicians’ offices.

Rather than ask a health care provider for advice, up to eight of the seventeen women first consulted their partner or a colleague about whether to seek health care. This finding emphasizes the importance of including partners, family members and others in the routine information given to all pregnant women about the signs and symptoms of PTL. The importance of including family members and/or friends in the provision of health information may be especially relevant for nulliparous women [21].

Women described arriving at the hospital for assessment of their symptoms feeling uncertain that they needed urgent care and expecting to be reassured that they were *not* in PTL. Rather than describing having to be seen at the birthing unit as anxiety provoking, women described feeling reassured by being assessed in a busy specialized unit. Women expressed confidence in the expertise of birthing unit staff and in the units’ system of prioritization according to urgency. Having to present oneself at the birthing unit for fFN testing was clearly acceptable and reassuring for the women in this study.

Women transitioned from feeling reassured to hoping for reassurance and beginning to feel anxious while they waited for their fFN results. This finding is interesting because stress and anxiety are known to be a serious concern for women already diagnosed with preterm labour. For example, Lowenkron reported that women with PTL experienced a moderate amount of stress, appraising their situation as threatening with negative connotations such as feeling frustrated, fear of possible bad outcomes and loss of control of their lives [24]. Mackey et al. found that women with PTL had significantly higher tension-anxiety and depression-dejection than those without PTL [25]. Our findings suggest that waiting for test results may be a key time for onset of women’s anxiety related to PTL that can be diminished with a clear explanation of the test and the ongoing presence of a support person. These findings are supported by studies with other populations that have compared levels of reassurance among patients provided with different amounts of information about diagnostic tests. For example, in a randomized controlled trial Petrie et al. [26] found that patients with chest pain who had a brief discussion with their physician about a diagnostic test, reported feeling more reassured than patients who received either the standard information or a pamphlet about the test. Similarly, in a recent systematic review of randomized controlled trials, the authors concluded that diagnostic tests alone do not reassure patients. They recommend providing clear explanations prior to diagnostic tests [27].

The participants’ descriptions of their fFN testing experiences often revealed that they felt increased anxiety while they waited for the results of their fFN screening, interpreted in our findings as moving from ‘feeling reassured’ to ‘hoping for reassurance’ or hoping for a negative test result. Although we cannot conclude from this qualitative study that women experience increased anxiety while waiting for their fFN results, this phenomenon is one that has been reported in other studies of women waiting for prenatal screening test results [28]. Bracing is a coping strategy commonly used by patients awaiting medical test results and is characterized by decreased optimism about their result. It is thought that bracing helps patients to prepare for a potentially poor result, and it is especially evident when there is a short waiting time between having the test and receiving the results. Examining the effect of bracing on individuals’ cognitive processing and recall of information, Portnoy [29] found that both are significantly diminished when individuals are waiting (bracing) for the results of a medical test. These studies provide theoretical support for our finding that the women in our study described increasing anxiety while waiting for the results of their fFN test. Implications for practice include consideration of the potential for women’s heightened anxiety and decreased recall while they are waiting for their fFN results. Potential interventions are to encourage women’s partners to stay during the waiting period, include them when providing information to women, and provide written information to enhance recall.

Upon receiving and understanding their fFN results, women with a negative result were initially reassured. However they subsequently articulated concerns about what remained unknown – if they were not in preterm labour then what *did* cause the symptoms that they experienced? Women with positive fFN results could no longer hope for reassurance from the test results and re-framed how they felt reassurance – the quick action and attention of their physician and the birthing unit team to address their PTL. Once they were stabilized, women with positive fFN results re-framed reassurance once again – as a need to know the cause of their PTL. These findings support those from other studies that, once diagnosed with PTL, women search for the cause of their PTL [19,20,30-32]. Our study findings add the understanding that women who are assessed in birthing units but determined not to be in PTL may experience similar anxiety related to not knowing the cause of their symptoms. Over time, and as some women are stabilized and discharged home with activity restriction, it may be that there is a continual process of re-defining reassurance. For example, community and family resources that relieve women of their home responsibilities may be experienced as reassuring with respect to their “work of keeping the baby in” [33]. When providing follow-up, providers can be reassuring by being considerate of some women’s “…profound sense of personal responsibility for preventing preterm birth…” (p. 65) and the guilt that some women feel when they do give birth prematurely [34]. The notion that women re-frame what they find reassuring provides guidance to health care providers in anticipating and providing timely interventions to reduce anxiety in women undergoing assessment for and living with PTL.

Fetal fibronectin testing was experienced by the women in this study as only one of many tests that they underwent after presenting to the birthing unit with symptoms of PTL. Nevertheless the process of fFN testing was experienced as being acceptable and valuable, in that women perceived the birthing unit to be a reassuring environment. In addition, whether fFN results were negative or positive, knowing and understanding the results contributed to women’s ability to reassure themselves. Based on these findings, it is unlikely that fFN testing increased women’s anxiety; rather it may play a role in an emotional process that reduces anxiety among women with symptoms of PTL. We have described this emotional process as one where women transition between seeking, feeling, hoping for and re-defining reassurance.

Future research employing a phenomenological approach is required to more fully understand the essence of women’s reassuring experiences when undergoing fFN testing for symptoms of PTL; or a grounded theory approach to more fully understand the emotional process of seeking reassurance experienced by women with symptoms of PTL.

### Strengths and limitations

One important strength of this study is the inclusion of a diverse group of women with respect to language (English and French), geography (urban and rural), parity and fFN results. Secondly, interviews were conducted by registered nurses with a thorough understanding of the clinical context and skills in qualitative interviewing.

The findings from this study should be interpreted cautiously as they are based on the experiences of a small group of women from one province in Canada. Furthermore, the women who participated in this study generally had a high level of social support, were well educated, and were mostly employed. While the results of this study may be transferable to settings with similar populations of women, future research should seek to examine and compare the fFN testing experience of women who are positioned differently (e.g. residing outside of Ontario, with less social support, education, and/or employment). Furthermore, the theme ‘feeling reassured’ described by the women in this study may not be entirely the result of fFN testing as they underwent multiple assessments and procedures as part of their assessment for PTL. However, we can conclude that fFN testing contributed to their experiences of ‘feeling reassured’. In particular, it is the availability of the fFN test that requires women be assessed in hospital, an environment that they described as contributing to being reassured. The remaining themes ‘hoping for reassurance’ and ‘re-defining reassurance’ are directly related to the process of waiting for and receiving fFN testing results.

## Conclusions

Our findings describe the experience of fFN testing from the perspective of study participants who presented to birthing units with symptoms of PTL. Women described being assessed for PTL as an emotional process that begins with expecting reassurance that they are *not* in PTL, feeling reassured by the birthing unit environment, hoping for reassurance from screening results, and re-defining reassurance upon being notified of their fFN results. The fFN testing process and results are components of care that provide important reassurance for women regardless of negative or positive results. The importance of reassurance for women experiencing symptoms of PTL should not be underestimated given the literature reporting the anxiety related to PTL symptoms [12,19,32]. These findings indicate the importance of providing comfort measures to minimize anxiety and increase reassurance during fFN testing, including clearly explaining test results to women and their partners.

## Abbreviations

fFN: Fetal fibronectin; NICU: Neonatal intensive care unit; PTB: Preterm birth; PTL: Preterm labour; RA: Research assistant.

## Competing interests

The authors declare that they have no competing interests.

## Authors’ contributions

WEP contributed to the design of the study, led the acquisition of data and data analysis, drafted and revised the manuscript. AES conceived of, and contributed to the design of the study, contributed to the interpretation of the data and critically revised the manuscript. JR participated in data collection and analysis, assisted in drafting and revising the manuscript. MW contributed to the design of the study, interpretation of the findings, and critically revised the manuscript. DBF, SLP, SID, and MJ all contributed to interpretation of the data and critically revised the manuscript. All authors read and approved the final manuscript.

## Authors’ information

WEP is a registered nurse and Associate Professor at the School of Nursing, Faculty of Health Sciences, University of Ottawa. AES is the Scientific Manager for BORN Ontario (Better Outcomes Registry & Network). JR is a registered nurse and research nurse at the Children’s Hospital of Eastern Ontario. MW is a maternal fetal medicine specialist & University of Ottawa, and Tier 1 Chair in Perinatal Epidemiology. DBF is an Epidemiologist with BORN Ontario (Better Outcomes Registry & Network). SLP is the Head of the Division of Biochemistry at The Ottawa Hospital and Associate Professor in the Departments of Pathology and Laboratory Medicine and Obstetrics and Gynecology at the University of Ottawa. SID is a researcher and the Knowledge Translation Specialist for BORN Ontario (Better Outcomes Registry & Network). MJ is a registered nurse and was responsible for coordinating the implementation of the fFN test across Ontario and currently works with BORN Ontario.

## Pre-publication history

The pre-publication history for this paper can be accessed here:

http://www.biomedcentral.com/1471-2393/14/190/prepub
